# Arsenic Contamination of Groundwater: A Review of Sources, Prevalence, Health Risks, and Strategies for Mitigation

**DOI:** 10.1155/2014/304524

**Published:** 2014-10-14

**Authors:** Shiv Shankar, Uma Shanker, Shikha

**Affiliations:** ^1^Babasaheb Bhimrao Ambedkar University, Lucknow 226025, India; ^2^Department of Chemistry, Dr. B. R. Ambedkar National Institute of Technology Jalandhar, Punjab 144011, India

## Abstract

Arsenic contamination of groundwater in different parts of the world is an outcome of natural and/or anthropogenic sources, leading to adverse effects on human health and ecosystem. Millions of people from different countries are heavily dependent on groundwater containing elevated level of As for drinking purposes. As contamination of groundwater, poses a serious risk to human health. Excessive and prolonged exposure of inorganic As with drinking water is causing arsenicosis, a deteriorating and disabling disease characterized by skin lesions and pigmentation of the skin, patches on palm of the hands and soles of the feet. Arsenic poisoning culminates into potentially fatal diseases like skin and internal cancers. This paper reviews sources, speciation, and mobility of As and global overview of groundwater As contamination. The paper also critically reviews the As led human health risks, its uptake, metabolism, and toxicity mechanisms. The paper provides an overview of the state-of-the-art knowledge on the alternative As free drinking water and various technologies (oxidation, coagulation flocculation, adsorption, and microbial) for mitigation of the problem of As contamination of groundwater.

## 1. Introduction

Contamination of groundwater, either from anthropogenic or natural sources with several social impacts, has now turned to be a major environmental concern in different parts of the world. Millions of people in several countries are exposed to high levels of As via intake of As-rich groundwater. Elevated level of As in groundwater has been well documented in Chile, Mexico, China, Argentina, USA, and Hungary [1, 2] as well as in the Indian State of West Bengal, Bangladesh, and Vietnam [2–6]. About 150 million people around the world are estimated to be affected globally with an increasing prospect as new affected areas are continuously discovered [7]. Arsenic, a well-known carcinogen, is considered as one of the world's most hazardous chemicals [8]. Excessive and long-term (such as 5–10 years) human intake of toxic inorganic As from drinking water and food may result in arsenicosis, a common name generally used for As related health problems including skin disorders, skin cancers, internal cancers (bladder, kidney, and lung), diseases of the blood vessels of the legs and feet, possibly diabetes, increased blood pressure, and reproductive disorders [9–11].

In terrestrial environment, the inorganic forms of As (such as trivalent arsenite (As^III^) and pentavalent arsenate (As^V^)) are more prevalent and toxic than the organic forms in general. As exerts detrimental effects on general protein metabolism with high toxicity by reacting with sulfhydryl groups existing in cysteine residues [12].

Arsenicosis causes dire consequences for the livelihood, family life, and earning capability when individuals fall victim. Deterioration in physical appearance makes women socially excluded. At larger perspectives elevated As contamination of a region may result in societal stress, disability in individuals, poverty, and decreased market value of potentially contaminated agricultural products leading to low income to the affected farmers [13]. Absence of taste, odour, colour, and exposure make As impossible for a layman to detect and avoid. Applying the WHO provisional guideline for drinking water of 10–50 ppb of As, a population of more than 100 million people worldwide is at risk, and of these more than 45 million people mainly in developing countries from Asia are at risk of being exposed to more than 50 ppb of As, which is the maximum concentration limit in drinking water in most of the countries in Asia [7].

At present, As is estimated to affect more than 150 million people worldwide with its increasingly elevated concentrations in drinking water [14]. The major arsenicosis affected areas have been reported in large deltas and/or along major river basins across the world [15] such as in Paraiba do Sul delta, Brazil [16], Bengal delta [17–19], Mekong delta, Cambodia [20], Danube river basin, Hungry [21], Hetao river basin, Mongolia [22], Duero Cenozoic Basin, Spain [23], Zenne river basin, Belgium [21], and Tulare Lake, USA [24]. The transfer of As to the food chain will ultimately remain as long-term risks to human and ecological systems [25]. Since water is the principal route through which As enters into the human body [26], the understanding of the processes of As contamination in groundwater, associated health risks, and mitigation of As problem is required.

The present review summarizes possible sources of As contamination of groundwater, global overview of groundwater As contamination, toxicity, basic chemistry, associated health risks, and the best available strategies for mitigation of As pollution in groundwater.

## 2. Sources, Speciation, and Mobility of As in Groundwater

Several natural and anthropogenic sources are deemed responsible for As contamination in groundwater. As occurs as a major constituent in more than 200 minerals [27] and the desorption and dissolution of naturally occurring As bearing minerals and alluvial sediments result in high As concentration in groundwater in deltas and alluvial plains even if the As concentration in the solid phase is not high [28, 29]. The presence of metalloid in excess concentration in groundwater may be associated with ore deposits where As is present predominantly in sulfidic minerals such as arsenopyrite and pyrite [30]. Arsenopyrite (FeAsS) is the most abundant As containing mineral generally existing in anaerobic environments and in various other rock forming minerals like sulfide, oxide, phosphate, carbonate, and silicate [1]. It is present as a substitute of S in the crystal lattice of various sulfide minerals. Realgar (As_4_S_4_) and orpiment (As_2_S_3_) represent the two common reduced forms of As while in arsenolite (As_2_O_3_), As is present in oxidized form [21]. Depending on the nature and texture of minerals, As can also be found in sediments, in the concentration range from 3 to 10 mg kg^−1^ [7]. The areas with high concentrations of Fe oxide or hydrous metal oxide or pyrites contain very high levels of As in sediments in comparison to other oxides. In reducing sediments, the concentration of As is found to be high; the concentration of As increases gradually with increase in the depth of the sediment [31]. Fe and Al oxides present in sediments play a significant role for the contamination of groundwater. Reductive dissolution of Fe and Al metal oxides along with the activity of indigenous metal reducing bacteria is now deemed as the prominent release mechanism of As, directly effecting the mobility of As [32, 33]. The main anthropogenic sources for contamination of groundwater with As are mining, burning of fossil fuels, use of arsenical fungicides, herbicides and insecticides in agriculture, and wood preservatives [21]. Burning of coal has profound effect on contamination of As in the environment. Emission of As takes place in the environment by volatilization of As_4_O_6_ due to burning of coal, which condenses in the flue system and ultimately transferred into water reservoirs [27]. The degree of groundwater arsenic contamination by aforesaid anthropogenic sources is much less as compared to the natural sources; however, their contribution cannot be neglected.

Arsenic in groundwater exists primarily as oxy anions representing two oxidation states: arsenic (arsenite) and arsenic (+V) (arsenate) [34, 35]. Arsenic in groundwater exists primarily as oxy anions representing two oxidation states: arsenic As^III^ (arsenite) and arsenic As^V^ (arsenate) [34, 35]. Both As^III^ and As^V^ exist within the pH range of 6–9. The predominant As^III^ species are uncharged H_3_AsO_3_ while the primary arsenate species are monovalent H_2_AsO^−4^ and divalent HAsO_2_^−4^. Geology and groundwater environment make one form, either As^III^ or As^V^ dominant [36, 37]. Although As^V^ is thermodynamically favored in oxic waters and As^III^ in anoxic waters, they have been also reported to coexist in both types of waters [38, 39]. Many researches of localized studies [40–43] have reported the value of arsenic speciation information in explaining and understanding the behavior and characteristics of arsenic in the environment (solubility, mobility, etc.). The toxicity and the removability of arsenic differ between As^III^ and As^V^. As^III^ is considered to be more toxic and more difficult to remove from water than As^V^ [8]. The variability of the arsenic concentration in groundwater is ascribed to the arsenic content of the aquifer and the varying dissolution/desorption processes releasing the arsenic from the solid phase into the liquid phase [36, 37, 44, 45]. Reductive dissolution of Fe oxides is considered as the principal cause of As release from aquifer sediments [46].

## 3. Global Overview of Groundwater Arsenic Contamination

The contamination of As can be propagated defectively into the groundwater system because As in groundwater and aquifers is mobilized (e.g., hydraulic fracturing). Hence, its contamination can affect a large population of people [47].

Groundwater concentration of As has been documented in the literature which reveals a very large range from less than 0.5 to 5000 ppb covering natural As contamination found in more than 70 countries [7]. Some of the best reported and most severe cases of arsenic contaminated groundwater have been found in aquifers across the globe which has been cited in Table 1. It represents that provisional guideline values for As concentration in groundwater are commonly set at 10 ppb, although it can reach up to 50 ppb. The outcomes of this comparison affirm that As contamination is a widespread global phenomenon and severe enough exceeding such guideline values. In fact, people consuming As-rich water for prolonged periods are reported to suffer from serious health problems in many parts of the world.

## 4. Arsenic: Health Risks

Arsenic contamination in the environment is turning to be a serious public health problem in several parts of the world. It is well-established fact that arsenite As^III^ is more toxic than arsenate As^V^, with inorganic As being more toxic than organic As [48]. However, different organic As species represent different degrees of toxicity. For instance, monomethylarsonic acid (MMA^V^) and dimethylarsinic acid (DMA^V^) as final As metabolites are less toxic than inorganic arsenic, whereas the degrees of toxicity of intermediate metabolites such as monomethylarsonous acid (MMA^III^) and dimethylarsinous acid (DMA^III^) are much more higher than inorganic arsenic. The toxicity of various arsenic species increases in the order of As^V^ < MMA^V^ < DMA^V^ < As^III^ < MMA^III^ ≈ DMA^III^ [48].

### 4.1. Uptake and Metabolism of Arsenic

**Figure 1:**
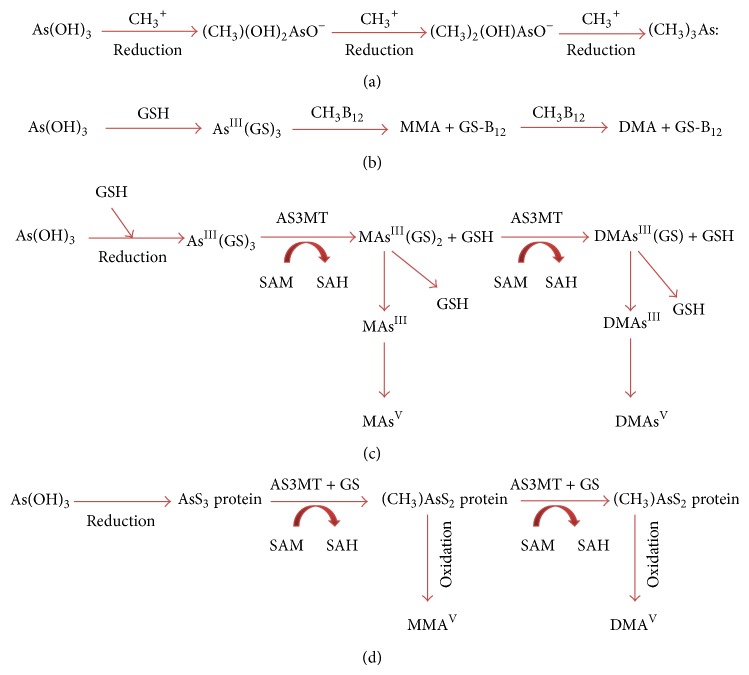
Pathways of arsenic metabolism in cells: (a) arsenic methylation in* Scopulariopsis brevicaulis* [54], (b) nonenzymatic As methylation in rat liver [55], (c) arsenic metabolic pathway in rat liver [56], and (d) metabolic pathway in rat liver [58], where SAM: S-adenosylmethionine; SAH: S-adenosylhomocysteine; CH_3_^+^: methyl group; GSH: glutathione; (CH_3_)(OH)_2_AsO^−^: monomethylarsonous acid; (CH_3_)_2_(OH)AsO^−^: dimethylarsinic acid; (CH_3_)_3_As: trimethyl arsine oxide; As (GS)_3_: arsenic triglutathione; MMA: monomethylarsonic acid; DMA: dimethylarsinic acid; MAs^III^ (GS)_2_: monomethylarsonic diglutathione; DMAs^III^ (GS): dimethylarsinic glutathione; DMAs^III^: trivalent monomethylarsonous acid; DMAs^V^: pentavalent dimethylarsinic acid; MMA^V^: pentavalent monomethylarsonic acid [224].

In terrestrial environment, As is mainly present as inorganic As, which exists as pentavalent (As^V^) under aerobic condition and trivalent (As^III^) under anaerobic environment [28]. As^III^ is generally found as a neutral species (As  (OH)_3_°, pKa = 9.2) in aqueous solution at physiological pH. As^III^ and As^V^ cause toxicity differently [49]. Due to its structural similarity to glycerol, As^III^ can be transported into cells through aquaglyceroporins, a pore protein for transporting small organic compounds such as glycerol and urea [50]. However, As^V^ takes different pathway into animals and human cells. As a phosphate analog, they have similar dissociation constants (pKa of arsenic acid: 2.26, 6.76, and 11.3 and pKa of phosphoric acid: 2.16, 7.21, and 12.3) [51]. Similar to phosphate, As^V^ is found in water as an oxy anions in solution, that is, H_2_AsO_2_^−^ and HAsO_2_^2−^ at pH 5–7. As chemical analogs, they compete for their entry via phosphate transporters [52]. Having entered into the human and animal cells, As^V^ is rapidly reduced to As^III^. Thereafter, As^III^ undergoes multisteps in cells through arsenite methyltransferase (AS3MT) using S-adenosylmethionine (SAM) as the methyl donor, resulting in the formation of methylated As compounds including MMA^III^, DMA^III^, MMA^V^, and DMA^V^ [53]. Challenger [54] first proposed the classical pathway of As methylation. He proposed that arsenic methylation involves a chain of oxidation and reduction steps (Figure 1(a)). Thereafter, Zakharyan and Aposhian [55] suggested that As^III^ can be methylated nonenzymatically in the presence of both methylcobalamin and glutathione (GSH) (Figure 1(b)). In several studies later on, researchers extensively explored the mechanism of arsenic methylation and concluded that the enzymes play crucial role in arsenic methylation. A new enzymatic metabolic pathway for arsenic methylation has been shown in Figure 1(c). The –OH groups of As (OH)_3_ are substituted by glutathionyl moieties, leading to the formation of GSH conjugates As (GS)_2_–OH and As (GS)_3_ [56]. Afterwards, as the major substrates for AS3MT, As^III^-glutathione complexes are further methylated to monomethylarsonic diglutathione MMA (GS)_2_ and dimethylarsinic glutathione DMA (GS). Since DMA (GS) is unstable, it is immediately oxidized to pentavalent DMA^V^, which is the major metabolite and is excreted from cells [57].

Naranmandura et al. [58] demonstrated a different pathway of arsenic metabolism via investigating the hepatic and renal metabolites of arsenic after an intravenous injection of As^III^ in rats (Figure 1(d)). They asserted that As^III^ confined to the proteins (AsS3 protein) is metabolized in the body at the time of successive reductive methylation by AS3MT in the presence of GSH and SAM and the reduced metabolites are excreted outwardly. Consistent with the mechanisms, both trivalent and pentavalent inorganic and organic arsenicals were detected in the urine of individuals after chronic exposure to arsenic and in cell culture medium followed by in vitro exposure to arsenic [59].

### 4.2. Toxicity of Arsenic

**Figure 2:**
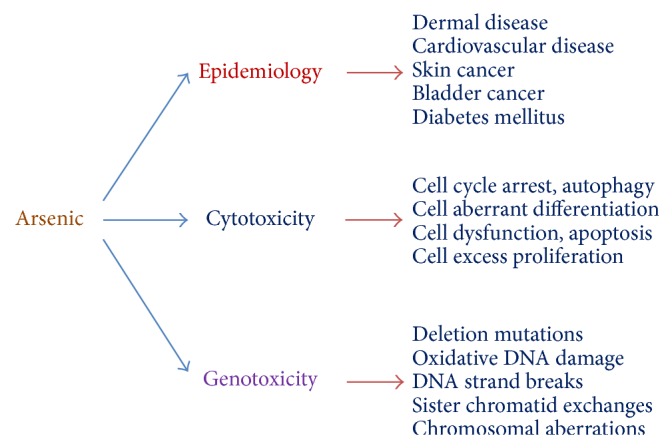
Arsenic toxicity in humans and rats [224].

Several review articles have documented arsenic toxicity in humans and animals (Figure 2). Arsenic is a potent carcinogen, leading to skin, bladder, liver, and lung cancers [60, 61]. Arsenic induces epidemiological toxicity. It results in the formation of excess ROS thereby damaging organisms [62, 63]. Arsenic is also known to cause cytotoxicity [64, 65] and genotoxicity [66, 67]. In addition, it is well-established fact that chronic exposure to arsenic can lead to arsenicosis, including skin lesions, blackfoot disease, peripheral vascular disease, and cancers. However, several studies have reported arsenicosis due to elevated level of As [68].

#### 4.2.1. Epidemiology

As a well-known human carcinogen, As-induced mechanism of carcinogenesis has been extensively explored in several studies. Mounting evidences have demonstrated that arsenic hinders a series of gene proliferation processes (e.g., DNA damage and repair, cell cycle, and differentiation) and distorts signal transduction pathways (e.g., protein 53 signaling pathway, Nrf2-mediated redox signaling pathway, and MAPK pathway) [69, 70]. ROS formation induced by As also plays a crucial role in triggering off cancer [71]. Further, investigations were of the opinion that methylation metabolites of arsenic are also potent carcinogens. Wei et al. [72] demonstrated that DMA causes cancer of urinary bladder in rats. Besides being well-known carcinogen, arsenic also causes a number of noncancerous multisystemic diseases including cardiovascular disease, dermal disease, hypertension, and diabetes mellitus [73, 74]. Researchers have pointed out that trivalent arsenicals (As^III^, MMA^III^, and DMA^III^) can induce diabetes via distortion of glucose metabolism based on intact pancreatic islets from mice [75, 76]. In addition, As led inhibition of pyruvate and *α*-ketoglutarate dehydrogenases has been found to be the principal cause of diabetes [77]. Cardiovascular diseases are closely linked with hypertension. There are several pathways for arsenic-induced hypertension, including promotion of inflammation activity and endothelial dysfunction, alteration of vascular tone in blood vessels, and malfunctioning of kidney [78]. In addition, several researchers were on the consensus that ROS's role in As-induced noncarcinogenic effects cannot be discounted [79, 80]. Arsenic-induced ROS has been correlated with alteration in cell signaling, apoptosis, and increase in cytokine production, leading to inflammation, which in turn results in formation of more ROS and mutagenesis, contributing to pathogenesis of arsenic-induced diseases [81].

#### 4.2.2. Cytotoxicity

Cytotoxicity develops when cell represents anomalies caused by toxic chemicals/contaminants. Arsenic led cytotoxicity in cells via several pathways has been explored by several researchers [82, 83]. Arsenic induces cytotoxicity by generating ROS [84]. ROS levels inside the cell increase dramatically when cell is exposed to elevated level of As. Arsenic results in ROS production by inducing NADPH oxidase [85]. Excess ROS causes damage in lipids and proteins as well as facilitating mitochondrial damage and its functions [86, 87]. Shen et al. [88] found that ROS-induced oxidative stress is caused by a mitochondria dependent apoptotic pathway. It has been well documented that ROS exerts cytotoxic effects by activating c-Jun N-terminal kinases (JNK), an important subgroup of the mitogen-activated protein kinases, which intercedes diverse cellular functions such as cell proliferation, differentiation, and apoptosis [89]. In addition, ROS can also act as modulators of signal transduction pathways, affecting various biological processes including cell growth, apoptosis, cell adhesion, and HIV activation [90, 91]. Arsenic results in cytotoxicity by affecting the status of tumor-suppressor protein 53 [92, 93]. Protein 53 plays a very crucial role in a wide range of cellular functions by modulation of transformation and regulation of cell growth and control of cell cycle, DNA synthesis, DNA repair and differentiation, and apoptosis [94, 95]. Yih and Lee [93] reported that arsenic may induce protein 53 accumulation in human fibroblasts, which ultimately results in cell apoptosis by promoting Bax translocation from the cytoplasm to the mitochondria, releasing cytochrome c and activating caspase-9 through Apaf-1 and the apoptosome [96, 97]. In addition, protein 53 can also result in inducement of cell cycle arrest at G2/M stage of the cell cycle by transcriptionally activating protein 21, the inhibitor of cyclin-dependent kinases [98, 99], and may induce autophagy in a DRAM (damage-regulated autophagy modulator) dependent manner [100].

#### 4.2.3. Genotoxicity

Damage of genetic information inside the cell results in genotoxicity which ultimately leads to mutation. Till date, there are several studies addressing the genotoxicity of As [101, 102]. As induces genotoxicity by generating ROS in similar fashion as cytotoxicity [103]. ROS present in excess amount within the cell reacts with cellular components and result in genotoxicity. Genotoxicity occurs since ROS reacts with both deoxyribose and bases in DNA, causing base lesions and strand breaks. In addition, ROS are also involved in oxidation of DNA, alteration of DNA repair, gene regulation mechanism, and threatening of gene stability [104]. As interacts with DNA repair proteins possessing functional zinc finger motifs, which are involved in transcription, DNA repair proteins, and DNA-protein and protein-protein binding [105, 106]. Zhou et al. [106] reported that As^III^ affects zinc fingers by binding with its target molecule PARP-1, ultimately leading to breaks of single-strand and double-strand of DNA and oxidative DNA damage [107]. Studies have found that arsenic can directly affect DNA repairing efficiency by lowering repair and expression of the nucleotide excision repair pathway member ERCC1 [108, 109]. Chronic exposure of cells to elevated level of arsenic can also result in induction of SAM depletion in cells, leading to loss of DNA methylation, and subsequently DNA hypomethylation in turn affects the genomic instability [110, 111]. Moreover, arsenic and trivalent methylated arsenic have been reported to interact with enzymes of SAM synthesis pathways [112, 113]. This is in agreement with Zhong and Mass [114] who affirmed that As^III^ or its metabolites can alter the activities of DNA methyltransferases and subsequently inhibit or stimulate the enzymes of SAM synthesis pathways. Similar to cytotoxicity, arsenic can also cause genotoxicity by affecting the status of protein 53 [115].

## 5. Mitigation of As Problem: Assessment of Technological Solutions

Selection of appropriate method to supply water with reduced As content relies on several factors and is complicated as the majority of the affected population lives in rural areas deprived off infrastructure and with decentralized water supplies from millions of shallow tube wells (STW) extracting water from shallow aquifers. Mitigation strategies for As contamination problem in groundwater therefore should address both technological and the socioeconomic considerations [116]. The various available options suited for getting drinking water with low As content can be divided into two categories which include

- finding an alternative As free water source,
- removal of As from the existing water source.

### 5.1. Alternative for Free Water Sources

#### 5.1.1. Deep Groundwater

Literature reviews from the Bengal and the Mekong deltas insinuate that As-rich water occurs mainly in the shallow groundwater, whereas groundwater from deeper aquifers is almost completely free from As. For example, the study by BGS revealed that only 5% of the deep tube well (>150 m deep) waters had As concentrations above 10 ppb and 1% exceeded the 50 ppb [117]; thus, water supply relying on manually operated deep tube wells (DTW) could be an appropriate source. However, the depth to As-free aquifers differs between the locations. For example, in the Bengal delta, generally water extraction below 150/200 m deep is considered as deep aquifer, but in many cases this can be below 200 m [118]. However, in the Red River and the Mekong deltas, As concentration is low at depths at only >50 m and >70 m [119]. The major restriction to the deep water extraction option is its costly installation, leading to its applicability only on community basis. Some other drawbacks to this option include availability of the As free deep aquifer, the uncertainty of the groundwater recharge mechanism [120], the risk of salt water intrusion in coastal areas, and very high concentrations of dissolved Fe and Mn [121]. Mn and Fe cause obnoxious taste and stains in water and laundry even at quite low concentrations exceeding 100 ppb. The permissible limit of Mn for same human health in groundwater for Mn is 400 ppb [9].

#### 5.1.2. Shallow Groundwater (Well Switching)

The As contamination in the shallow groundwater varies greatly [1] and the countywide study by British Geological Survey in Bangladesh [117] and as reviewed by Chakraborti et al. [122] reported that in Ganga-Meghna-Brahmaputra plain, the proportion of As contamination in tube wells is in the range of 20% to >50% and, hence, it is often possible to get uncontaminated tube wells in many areas within reasonable distances and well switching to an uncontaminated shallow tube well can be a suitable option. Among the various tried mitigation strategies, well switching to shallow tube wells has been found as most preferred strategy (29%) [123]. The major drawback to well switching option is the degree of the spatial and temporal variation in As level in groundwater, making it difficult, unpredictable for its reliably. Further studies reveal that the As concentration in the tube wells changes over time, and it is high during the monsoon period as compared to dry winter season [124, 125]. This means that the monitoring of each and every well may be required and, furthermore, long-term analysis is equally needed to guarantee that the tube wells will remain As free.

The other factors making this option unconvincing could be socioeconomic barriers, because well switching results in potential usage of other tube wells where entry may be restricted or barred in case of well being privately owned.

#### 5.1.3. Dug Well Water

By constructing open wells, generally called dug wells (DWs) with large diameters, As free safe drinking water can be obtained from As contaminated shallow aquifers. DWs used to be one of the alternative sources of water supply in the Bengal delta, before the installation of tube wells [126]. Studies have shown that the As level in most of the DWs is very low [126–128] due to prevailing oxidative environment and precipitation of Fe or due to groundwater recharge of the DWs with rainwater with few exceptions [129]. DWs have been suggested as the preferable alternatives of safe drinking water by The National Policy for As Mitigation in areas marked with high concentration of As in Bangladesh [130]. The evaluation of dug well performance in early stages of implementation establishes that these options are appropriate [131, 132], although prolonged studies report that tube wells will be the preferred choice over DWs [133, 134].

The reasons for the unpopularity of the DWs are obnoxious smell and taste, turbidity, and distance and time bound limitations to fetch water [131, 133]. Bacteriological contamination is the principal problem associated with the use of DWs water. The use of drinking water from these sources without appropriate treatment may lead to diseases like diarrhea, dysentery, typhoid, cholera, and hepatitis. The frequency of microbial contamination of DWs with thermotolerant coliforms (TTC) has been found as high as 94% with seasonal variation with higher contamination during the monsoon compared to the dry season [135]. New DWs usually have high bacterial contamination, which can be regulated by initial or even repeated disinfection like chlorination [129].

(*1) Surface Water*. Ponds, lakes, and rivers are generally low or free of As and can be reintroduced in affected areas as a source of safe drinking water. Most of the As affected areas are in the vicinity of a large river and these rivers can serve as sustainable mitigation option for long run, that is, over decades. Similar to the DWs, the major and critical limitation of using ponds and lakes is the risk of potential bacteriological contamination which was also the main reason behind replacement of surface water with groundwater as the drinking water source. Reintroduction of surface water as a source of safe drinking water would require antimicrobial treatment like incorporation disinfectants, use of pond sand filters (PSF) [136], or combined surface water treatment units. The use of PSF is preferred by The National Policy for As Mitigation for its application in As-affected regions in Bangladesh [130]. About 95% PSFs have been found microbially contaminated with elevated levels of TTC in the monsoon season as compared to the dry season [135].

(*2) Rainwater Harvesting*. Since ancient times, the rainwater harvesting (RWH) has been widely used practice throughout the world as a potential method of utilizing rainwater for domestic water supply [9]. RWH is widely used method at household level globally and there is also an increasing trend on its application at larger community level. The rainwater is safe if it is hygienically maintained and this technology is feasible in areas with average rainfall of 1600 mm/year or more [137]. RWH is among one of the preferred choices by The National Policy for As Mitigation for use in As-affected areas in Bangladesh [130]. In coastal areas, rainwater is the main source of drinking water because of the high salinity in shallow and deep tube wells. In these areas, rainwater is preserved in large ponds, [138] and the experience from such areas can be transferred to other As affected areas. One of the critical limitations of grass root implementation of rain water harvesting technology is its high installation cost in the form of special roofs and large storage tanks for collection and storage of rain water [126] due to the unequal distribution of rainwater over the year. Microbial contamination is also another limitation [138, 139] which can be avoided by discarding the rainwater collected from first flush [9]. Immense care should also be taken on the materials that come in contact with rainwater (especially zinc and lead), as rainwater is slightly acidic and can result in dissolution of metals and other impurities from materials of the catchment and storage tank, leading to unacceptably high concentrations of contaminants in the water [9, 135].

### 5.2. Removal of Arsenic

Removal of As mainly depends on the composition and chemistry of the As contaminated water. As occurs as As^III^ in most of the major reported cases and oxidation of As^III^ to As^V^ is considered as necessary to obtain satisfactory As removals.

#### 5.2.1. Oxidation

The main aim of oxidation is to convert soluble As^III^ to As^V^, which is followed by precipitation of As^V^. This is essential for anoxic groundwater, since As^III^ is the prevailing form at near neutral pH [140]. As^V^ adsorbs more freely onto solid surfaces than As^III^ and, thus, oxidation followed by adsorption is deemed to be effective for the removal of As [141, 142]. Several oxidants have been utilized for the oxidation. The kinetics of the reaction with O_3_, H_2_O_2_, Cl_2_, NH_2_Cl, and ferrate are of first order reactions with reference to both As^III^ and oxidants and, thus, the concentrations of As^III^ and the oxidant are the critical parameters for effective removal of As from aqueous solution. The reaction is very fast for permanganate, chlorine, and ozone as compared to hydrogen peroxide and chloramine when applied for oxidation of As^III^ to As^V^ [142–144]. Bajpai and Chaudhuri [145] reported that 54–57% of As^III^ can be oxidized to As^V^ in contaminated groundwater using air and pure oxygen whereas complete oxidation of As^III^ can be obtained with ozone. Manganese dioxide polished sand is another oxidant, with the advantage of being both an oxidizing agent as well as an adsorbent. The application of manganese dioxide polished sand is more effective when it is coupled with Fe containing compounds as the treated products are more filterable and easy to handle [145]. Recently, Criscuoli et al. [146] studied the oxidation of As^III^ by MnO_2_ coated PEEC-WC nanostructured capsules and demonstrated that they possess a higher efficiency than conventional oxidation methods when the water contains a low level of As. More than 99% oxidation was obtained at 100 to 300 ppb of initial As concentration. However, increasing the concentration to 700 and 1000 ppb, only 90% and 73% of As^III^ were oxidized, indicating a decreased oxidation efficiency of the particle with increase in the initial As^III^ concentration.

Photochemical and photocatalytic oxidation of As^III^ has also been explored in several investigations. In water, the rate of oxidation of As^III^ can be increased by UV radiation in the presence of oxygen. UV/solar light facilitates the generation of hydroxyl radicals through the photolysis of Fe(III) species (FeOH_2_^+^) and in presence of both hydroxyl radicals and O_2_, the rate of oxidation becomes faster [147, 148]. In addition, oxidation of As^III^ to As^V^ by photocatalytic oxidation and TiO_2_ followed by adsorption of As on TiO_2_ has also been investigated [149, 150].

A TiO_2_ coated chitosan bead (TICB) was synthesized by Miller and Zimmerman [151] and was applied for oxidation as well as removal of As from aqueous solution. They observed a higher amount of adsorption of As (6400 *μ*g As^III^g^−1^ TICB and 4925 *μ*g As(V)g^−1^) followed by UV radiation as compared to the solution that was not exposed to UV light (2198 *μ*g As (III)g^−1^ TICB and 2050 *μ*g As(V) gl^−1^). Their study concluded that the surface area of the TICB was increased and TiO_2_ was able to photooxidize As^III^ to As^V^ in presence of the UV light. In another investigation, [152] synthesized nanocrystal line Al_2_O_3_ and TiO_2_ impregnated chitosan for the removal of As. The study suggested a mechanism wherein As^III^ is photooxidized to As^V^ by TiO_2_ and is subsequently adsorbed by Al_2_O_3_. However, further research is required to implement this system at grass root level. Furthermore, the initial As concentration, pH, natural organic matter (NOM), and the presence of anions essentially affected the rate of adsorption of As(V)on TiO_2_ [150, 153–155]. The TiO_2_/UV system has an inefficient removal of As because of incomplete oxidation of As^III^ to As^V^ when a trace of TiO_2_ is present [156]. Presence of interfering substances such as Fe(II), Mn(II), sulfide (HS^−^ and S^2−^), total organic carbon (TOC), and dissolved organic carbon affect the oxidation of As^III^ in water samples. In the presence of S^2-^ and TOC, the oxidation rate of As^III^ by ozone severely declines [144].

#### 5.2.2. Coagulation-Flocculation

The incorporation of a coagulant followed by the formation of a floc is a potential method used to remove As from groundwater. Positively charged cationic coagulants decrease the negative charge of the colloids and, consequently, larger particles are formed due to aggregation of particles [157]. The flocs formed in flocculation process are because of polymeric bridging between the flocculent particles which later agglomerate to form larger mass particle. Soluble As is precipitated/coprecipitated onto the flocs and thus eliminated from aqueous solution. For As removal, Fe and Al based coagulants are widely used [158] among the various chemical coagulants. Pallier et al. [159] used kaolinite and FeCl_3_ as a coagulant/flocculent and they obtained more than 90% and 77% removal of As^V^ and As^III^, respectively, using 9.2 ppm of Fe^3+^. Recently, Hu et al. [160] used three aluminum based coagulants (aluminum chloride and two types of poly aluminium chloride) and all of them were found to reduce the concentration of As below the MCL with an initial As^V^ concentration of 280 ppb. Their study asserted that the aluminum species regulate the removal of As and thus As removal efficiency can be improved by adjusting the pH. Bilici Baskan and Pala [161] optimized the effects of major operational parameters such as the initial As^V^ concentration, the coagulant dose, and pH and achieved 91% removal of As^V^ with an initial As^V^ concentration of 10 ppb and an Al_2_(SO_4_)_3_ coagulant concentration of 66 ppb, and a removal of nearly 100% with an initial As^V^ concentration of 500–1000 ppb and a coagulant concentration of 42–56 ppb. Fe based coagulants have also been investigated by several authors [162–165]. Among the chemical coagulants, Fe based coagulants have been found to be efficient in treatment of water than the Al based coagulants [7]. For efficient removal, the As requires to be adsorbed on the amorphous metal hydroxides formed from the coagulant. However, the critical limitation of the coagulation/flocculation process is the production of a huge amount of sludge with a considerable concentration of As. The management of the contaminated sludge is important for safeguarding the environment from secondary pollution and thus reduces the applicability of this method in field conditions.

#### 5.2.3. Adsorption

Removal of As by adsorption onto activated/coated surfaces is getting popular because of its simpler operation and sludge free day to day operation. Several of the adsorbents can be regenerated and reused which is an extra advantage of this technology. Mohan and Pittman [166] reviewed more than 40 different types of adsorbents with more than 500 literature references. In addition, there is an increasing interest in exploration and improvement in new adsorbents. However, excluding few adsorbents like activated alumina and granulated ferric hydroxide, the information about most of the adsorbents is restricted to laboratory evaluations. The removal of As by adsorption techniques in general depends on pH and the speciation of As with better As^V^ removals as compared to As^III^ at pH lower than 7 [167–171]. Lin and Wu [172] reviewed that the rate of As adsorption and capacity adsorbents further depends on presence of other ions like phosphate, silicate, HCO^−3^, and Ca^2+^ competing for the adsorption sites. Zhu et al. [170] and Kanematsu et al. [171] also substantiated this fact. The most widely tested aluminium oxide is activated alumina (AA) [172–174] and is prepared by thermal dehydration of aluminium hydroxide. Various pretreatments like impregnation with Fe [173, 175], alum [176], manganese acetate [177], and posthydrolysis [177] have also been explored at lab scale to improve the adsorption efficiency of AA with promising results. Field experience is well documented from various studies, for example, Bamwsp et al. [178, 179]. Ferrihydrite, granular ferric hydroxide, and hydrous ferric oxide are the most widely explored iron oxides and hydroxides for the removal of As yielding promising results for both As^III^ and As^V^ removals [174, 180–184]. One of the major problems encountered on the field by aforesaid adsorption methods is the presence of high iron content in groundwater, which emanates into clogging of the filter material thereby reducing the lifetime of the filter [178, 185]. In the last decade, removal of As using zero valent iron (ZVI) or Fe(0) for removal of As has been widely explored by several research groups both in the laboratory [142, 186, 187] and in the field [188–192]. The removal mechanisms for As and other contaminants using ZVI have been reviewed by Noubactep in great detail [193]. According to Hussam and Munir [190], approximately 350,000 ZVI filters are operational in Bangladesh, Nepal, Pakistan, India, and Egypt and there are several studies showing promising results of As removals in field [188–192]. However, filters should be maintained properly; otherwise, they are clogged and not reliable in removing As [134].

latest advancements on arsenic removal by sorption.

#### 5.2.4. Latest Advancements on Arsenic Removal by Adsorption

A wide spectrum of different materials have been explored for adsorption of arsenic from groundwater water but iron oxides and oxyhydroxides are the most widely studied and their commercial products already dominate a major portion of the market [166, 194]. In water treatment plants, iron oxyhydroxides are used as mechanically resistant particles in fixed-bed pressure columns. The application of iron oxyhydroxides is encouraged due to their cheap and easy production. The amorphous structure of such hydroxides provides high specific surface area values and their strong affinity and relative high selectivity for the most frequently occurring arsenate species under natural pH-values of potable water.

Tresintsi et al., 2012 [194], synthesized various iron oxyhydroxides between the pH range 3–12 using the most common low cost iron salts (FeSO_4_ *·*H_2_O and FeCl_2_ *·*H_2_O) in a continuous flow kilogram-scale production reactor under intense oxidative conditions to serve as arsenic adsorbents. Synthesized iron oxyhydroxides at acidic (pH 4.0) and highly oxidizing conditions resulted in a very effective arsenic adsorbent comprising of uncrystallized schwertmannite. The high As^V^ sorption capacity of hydroxides was mainly determined by the reaction parameters controlling the effective surface charge and the positive role of adsorbed sulfates in the ion exchange with arsenate oxyanions.

The optimized adsorbent demonstrates the highest reported adsorption capacity while keeping the residual arsenic level below 10 mg/L (Q10-value) and maintaining its superiority in column investigations as compared to commercial granular materials. This method is simple and economically viable synthetic method adapted in a continuous flow production and a promising technology for scaling up.

Zhang and Sun, 2013 [195], invented multifunctional micro/nanostructured MnO_2_ spheres successfully and applied them in the removal process of As species from groundwater. Batch experiments revealed that As^III^ species can be effectively oxidized by the synthesized MnO_2_ followed by the adsorption of As^V^ species. Experimental results of this study insinuated that the synthesized material is repudiated with good adsorption and oxidative capacity required for the removal of arsenic species under controlled conditions. In addition, the synthesized MnO_2_ spheres can be efficiently recovered for their reuse by a microfiltration process with limited membrane pore blocking owing to the microstructure of the material. Synthesized MnO_2_ spheres are multifunctional materials with good oxidation, adsorption, and separation properties and can be utilized for water purification.

Cui et al., 2013 [196], synthesized highly porous, nanostructured ZrO_2_ spheres from amorphous ZrO_2_ nanoparticles with the help of a food-safe additive, agar powder, which yielded a simple, cheaper, and safer process for the synthesis of ZrO_2_ spheres. These ZrO_2_ spheres displayed good adsorption capacity on both As^III^ and As^V^ at near neutral pH, without the requirement of preoxidation and/or pH adjustment of the arsenic contaminated water. These ZrO_2_ spheres are highly stable, nontoxic, acid-alkali resistant and with high arsenic adsorption capacity. These ZrO_2_ nanoparticles seem to be prospecting material for their promising application in removal of arsenic from groundwater.

Cui, 2014, [197] conducted batch and continuous-flow pilot investigations employing ultrasound (US), ultraviolet (UV), and a combination of US and UV to gauze the rate of oxidation of arsenite (As^III^). As compared to the single processes of US or UV, the combined US/UV system proved to be the best for As^III^ oxidation with a synergy index of more than 1.5. A high rate constant of As^III^ removal was achieved as ferrous [Fe(II)] ions existed. As an energy-utilizing oxidation technique does not require a catalyst, the combined energy system employing US/UV followed by MF could be a promising alternative for treating As^III^ and Fe(II) simultaneously.

#### 5.2.5. Biological Arsenic Removal: Basic Techniques

Bacteria play crucial role in geochemical cycling of As by oxidation/reduction reactions, determining its speciation and mobility [1]. Arsenic pentavalent (As^V^) reduction and arsenic trivalent (As^III^) oxidation are both detoxification mechanisms [198]. Bacterial species coupling anaerobic oxidation of organic substrates to the reduction of arsenates have also been reported by several researches. Such bacteria are known as dissimilatory arsenate reducing bacteria or arsenate respiring bacteria (ARD), for example,* Geospirillum arsenophilus*,* Geospirillum barnesi*,* Desulfutomaculum auripigmentum*,* Bacillus arsenicoselenatis,* and* Crysiogenes arsenatis* [199–202]. These bacteria use As^V^ as a terminal electron acceptor in their respiratory process. The oxidation of As^III^ is generally carried out by the incorporation of chemical reagents such as ozone, hydrogen peroxide, chlorine, or potassium permanganate [203–205]. The use of chemical reagents in drinking water treatment is discouraged as it often leads to the formation of undesirable byproducts such as trihalomethanes (THMs) [206, 207].

Biological oxidation of As^III^ can be applied as an alternative to the chemical oxidation. Iron and manganese are typical unwanted constituents in drinking water causing aesthetic problems known to play significant role in arsenic concentrations in groundwater. Several species of bacteria have been reported to carry out biological oxidation of As [35, 208, 209]. Specific indigenous bacteria mediating biological oxidation of arsenic are known as “iron and manganese-oxidizing bacteria.” These bacteria have been successfully applied for the biological arsenic oxidation directly in continuous groundwater treatment [210–212].

The biological oxidation of iron by two bacteria,* Gallionella ferruginea* and* Leptothrix ochracea,* has been found to be a promising technology for effective removal of arsenic from groundwater [213]. In this process, iron oxides are coated on filter medium, along with the microorganisms, which offer an ideal environment for arsenic to be adsorbed and removed from the water. Under optimum experimental conditions, trivalent arsenic has been found to be oxidized by these bacteria, contributing to almost complete arsenic removal (up to 95%) even when initial arsenic concentrations were 200 mg/L [213]. The pentavalent arsenic content, under the aforesaid experimental conditions, can be removed significantly, leading to residual concentrations below the newly enforced limit of 10 mg/L. This technology efficiently removes arsenic from groundwater and offers several advantages as compared to conventional physicochemical treatment processes. It avoids the incorporation of chemical reagents for the oxidation of trivalent arsenic; therefore, it is a cost effective and eco-friendly option. In addition, it does not need monitoring of a breakthrough point, as in various sorption processes, because the sorbents (iron oxides) are consistently produced* in situ*. Due to being a combined treatment process (biological oxidation, filtration, sorption process), it can be used for the simultaneous removal of other inorganic contaminants such as iron, manganese, and arsenic from groundwater [213].

Katsoyiannis et al., 2008, [214] demonstrated the application of a treatment method for the removal of iron, ammonium, manganese, and phosphate from groundwater. In this method, the biological oxidation of ammonium and Mn(II) for the simultaneous As^III^ oxidation and subsequent As^V^ removal by coagulation from groundwater was applied. This method is a combined groundwater treatment approach, that is, bioremediation coupled with physicochemical treatment with low operational costs [214]. A water treatment unit based on this technology is operational in northern Greece in the city of Malgara. The same can be effectively applied at grass root level in other arsenic contaminated parts of the world.

Katsoyiannis et al. 2013 [215] studied removal of As^III^ and As^V^ from groundwater by the application of biological oxidation of dissolved iron and manganese in a pipe reactor (PR), followed by microfiltration (MF). The groundwater under test (Berlin, Marienfelde) contained average Fe(U) and Mn(II) concentrations of 2.9 and 0.6 mg/L, respectively. Oxidation of these metals imparted adequate adsorption sites and, therefore, arsenic species could be removed from groundwater effectively. The residual concentrations in all cases were found to be reduced up to 10 *μ*g/L. The initial concentration of arsenic in water was in the range of 20 to 250 *μ*g/L.

Advantageous aspect of this technology is the uptake of oxidized iron and manganese onto recirculated suspended solids which flocculated in the pipe reactor, thereby eliminating the requirement for mechanical cleaning of the membrane, while keeping the transmembrane pressure (TMP) constantly low. The As^V^ removal capacity of this hybrid PR-MF unit was found to be significantly higher than that achieved by conventional coagulation-filtration with Fe(III). Conclusively, the very latest PR-MF process efficiently removes iron, manganese, and arsenic without using chemical reagents for oxidation or pH adjustment, and without the requirement of regular regeneration or backwashing, and thus it follows the principles of green chemistry [215].

## 6. Conclusion

Arsenic contamination of groundwater is an alarming problem on a global scale. In several parts of the world, biogeochemical processes have resulted in dissolution of naturally occurring As into groundwater. In present review, we tried to elaborate on different natural and anthropogenic sources of As in groundwater including its speciation and mobilization pattern in groundwater. We have also reviewed problem of As contamination in groundwater in different parts of the world followed by detailed outlook in epidemiology and toxicity mechanisms of As in animals and humans. In order to combat arsenic problem, various remediation methods based on conventional, modern, and hybrid technologies for removal of As in several parts of the world have been critically reviewed. The merits and demerits of these technologies have been discussed in detail. Most of the existing technologies for removal of As involve the direct removal of As^V^ or converting As^III^ to As^V^ followed by removal of As^V^. The implementation of mitigation options can be facilitated by setting proper guidelines and to control implementation at appropriate intervals. The awareness of the population is deemed equally important in maintaining and choosing mitigation. However, even for well-aware population, the dilemma is often the ability to meet prohibitive costs versus the wish to improve their situation. For communities public participation encounters the same constraints. Governmental and donor financial and logistic assistance may be essential to reduce arsenicosis. Besides, extensive research should address the understanding of the occurrence, origin, and distribution pattern of arsenic. The government should monitor industrial and agricultural activities leading to As pollution. More technical assistance should be rendered to mining or chemical plants to deal with sewage and sludge storage and waste treatment. Supervision departments should increase the frequency of sampling and analysis of the discharge from industrial plants. We sincerely hope that this paper will be of considerable interest to the readers. The paper reflects the latest state of the art on understanding of various interdisciplinary facets of the problem of arsenic in environmental realm, mechanisms of mobilization in groundwater, biogeochemical interactions, and the measure for remediation.

## Figures and Tables

**Table 1:** Status of As contamination in natural groundwater in various countries.

Serial number	Country	Region	Groundwater As level (ppb)	Permissible limit (ppb)	References
1	Afghanistan	Ghazni	10–500	10 (WHO)	[21]
2	Australia	Victoria (around the gold-mining regions)	1–12 (Groundwater); 1–73 (Drinking-water); 1–220 (Surface water)	—	[17, 21, 216]
3	Bangladesh	Noakhali	<1–4730	50 (WHO)	[18, 21, 217]
4	Brazil	Minas Gerais (Southeastern Brazil)	0.4–350 (Surface water)	10 (WHO)	[17, 216, 218]
5	Cambodia	Prey Veng and Kandal-Mekong delta	Up to 900 1–1610	10 (WHO)	[20, 21]
6	Canada	Nova Scotia (Halifax county)	1.5–738.8	10 (WHO)	[17, 21]
7	China	—	50–4440	50 (WHO)	[219]
8	Finland	Southwest Finland	17–980	10 (WHO)	[17, 216, 218]
9	Greece	Fairbanks (mine tailings)	Up to 10,000	10 (WHO)	[1, 21]
10	India	West Bengal Uttar Pradesh	10–3200	50 (WHO)	[1, 21, 216, 220, 221]
11	Japan	Fukuoka Prefecture (southern region)	1–293	10 (WHO)	[17, 216]
12	Mexico	Lagunera	8–620	25	[1, 21, 216]
13	Nepal	Rupandehi	Up to 2620	50	[21, 219, 222]
14	Pakistan	Muzaffargarh (southwestern Punjab)	Up to 906	50	[17, 22, 216]
15	Taiwan	—	10–1820	10 (WHO)	[1, 21, 216]
16	Thailand	Ron Phibun	1–>5000	10 (WHO)	[1, 21, 216]
17	USA	Tulare Lake	Up to 2600	10 (USEPA)	[21, 24, 223]
18	Vietnam	Red River Delta (Northern Vietnam) Mekong Delta (Southern Vietnam)	<1–3050	10 (WHO)	[1, 219]

## Abbreviations

As: Arsenic
${\text{As}}^{\text{III}}$: Arsenite
${\text{As}}^{\text{V}}$: Arsenate
${\text{MMA}}^{\text{V}}$: Monomethylarsonic
${\text{DMA}}^{\text{V}}$: Dimethylarsinic
${\text{MMA}}^{\text{III}}$: Monomethylarsonous
${\text{DMA}}^{\text{III}}$: Dimethylarsinous
AS3MT: Arsenite methyltransferase
SAM: S-Adenosylmethionine
As(GS)_2_–OH, As(GS)_3_: Arsenite-glutathione complex
GSH: Glutathione
ROS: Reactive oxygen species
DRAM: Damage-regulated autophagy modulator
ppb: Parts per billion
ppm: Parts per million,
DMA(GS): Dimethylarsinic glutathione
DWs: Dug wells
MCL: Maximum contaminant level
ERCC1: Excision repair cross-complement.
